# ISPAD and its role in the management of diabetes in the young in the Indian subcontinent and the Far East

**DOI:** 10.1186/1687-9856-2015-S1-O15

**Published:** 2015-04-28

**Authors:** Stephen Greene

**Affiliations:** 1 University of Dundee, Dundee, Scotland, UK

## 

Over the last three decades, while the fundamental need for insulin replacement in Type 1 diabetes (T1D) in young people remains, the method of delivery has changed dramatically.

From the “First World” view, “intensive insulin” therapy offers the best outcome, in terms of glycaemia and long-term health, and a shift to multiple injection therapy and pumps has become the approach promoted from diagnosis. A recent recognition is the sustained effect of near-normal glycaemia from diagnosis (‘metabolic memory’), achieved through strict glucose targets and dose adjustment of insulin for carbohydrate.

To use the developing technology of T1D requires considerable motivation from patients and their families and there is a need for a parallel support programme from a multidisciplinary team. The components of successful adherence to the management regimens are a matching of health beliefs, attuned communication and reciprocity between those with diabetes, the families and their health professionals.

The regions of the Indian Subcontinent and the Far East offer a significant challenge for ISPAD, an organisation dedicated to support education, science and advocacy for diabetes in the young. The heterogeneity of health care services and social standing of the component countries is significant test for ISPAD. The Society has risen to this test though a portfolio of activities, projects and administrative approaches, to work in partnership with organisations in the region dedicated to improving the outcome of diabetes in the young.

